# Porous Silicon Bragg Reflector and 2D Gold-Polymer Nanograting: A Route Towards a Hybrid Optoplasmonic Platform

**DOI:** 10.3390/nano9071017

**Published:** 2019-07-16

**Authors:** Paola Pellacani, Lucia Fornasari, Chloé Rodriguez, Vicente Torres-Costa, Franco Marabelli, Miguel Manso Silván

**Affiliations:** 1 Plasmore S.r.l., Via Riviera 12b, 27100 Pavia, Italy; 2 Department of Applied Physics and Instituto Nicolás Cabrera, Universidad Autónoma de Madrid, Campus de Cantoblanco. C/Francisco Tomás y Valiente, 7, 28049 Madrid, Spain; 3 Centre for Micro Analysis of Materials, Autonomous University of Madrid, 28049 Madrid, Spain; 4 Department of Physics, University of Pavia, Via Bassi 6, 27100 Pavia, Italy

**Keywords:** plasmonic nanograting, porous silicon, distributed Bragg reflectors, hybrid photonic–plasmonic devices, optoplasmonic platforms, biosensing

## Abstract

Photonic and plasmonic systems have been intensively studied as an effective means to modify and enhance the electromagnetic field. In recent years hybrid plasmonic–photonic systems have been investigated as a promising solution for enhancing light-matter interaction. In the present work we present a hybrid structure obtained by growing a plasmonic 2D nanograting on top of a porous silicon distributed Bragg reflector. Particular attention has been devoted to the morphological characterization of these systems. Electron microscopy images allowed us to determine the geometrical parameters of the structure. The matching of the optical response of both components has been studied. Results indicate an interaction between the plasmonic and the photonic parts of the system, which results in a localization of the electric field profile.

## 1. Introduction

The fascinating properties of photonic and plasmonic systems have served as ingredients in many different fields where an improvement of the light manipulation efficiency is required, such as in photovoltaic cells and biochemical sensing devices.

Metal-dielectric interfaces are known to guide Surface Plasmon–Polariton (SPP) modes, while nanometric metallic structures support Localized Surface Plasmons (LSPs). The recent and rapid development of research in this area was catalysed when scientists realized that SPP and LSP modes may lead to the localization of guided light signals far beyond the diffraction limit for electromagnetic waves in dielectric media. Various types of metallic nanostructures have been proposed and explored since then [1,2,3]. In particular, plasmonic nanogratings attracted interest for their transmission enhancement behaviour [4,5,6,7,8]. Plasmonic gratings can confine and enhance the electromagnetic field and have a significant application in the context of plasmonic photovoltaics [9,10,11,12] and in biosensing platforms [13,14]. Several studies have been devoted to investigate, both experimentally and theoretically, the optimization of the gratings design to enhance their sensing capability [15,16,17]. 

In the last decade, a plasmonic nanograting system constituted by a lattice of polymeric pillars embedded in a gold matrix has been developed [18]. In this type of system the electromagnetic field is strongly localized at the top of the polymeric pillars, making this surface particularly attractive for biosensing [19,20]. Moreover the soft processing conditions of the fabrication protocol (colloidal lithography and plasma techniques) offer the possibility of integrating these nanostructured plasmonic structures onto complex substrates (such as distributed Bragg reflectors (DBR), Organic Light Emitting Transistors and Diodes), in order to obtain miniaturized, portable and low energy consuming sensing devices. It is however worthwhile noticing that, although plasmonic systems enable the concentration of light below the diffraction limit, their quality factor Q is constrained by large radiative and dissipative losses.

Photonic Crystals (PCs) are another important means to manipulate and modify light propagation. A PC is a composite dielectric structure, which presents a spatial periodic modulation of the dielectric function with a period of the order of the wavelength range under consideration. A PC exhibits a photonic band gap, which prevents the propagation of light within a certain frequency range [21]. Differently from plasmonic structures, PCs can generate high quality factor resonances by having a diffraction-limited modal volume. 

Compared to other photonic structures, PCs made of porous silicon (PSi) show a very high potentiality due to their optical versatility and ease of fabrication [22]. The porosity and thus the refractive index of PSi can be accurately tuned by controlling the current density applied during its electrochemical etch [23]. The current density can be varied over time to tailor a desired refractive index profile along the PSi etch pathway. This approach has enabled the fabrication of elements such as DBRs [24,25], optical filters [26,27], vertical micro-cavities [28], and three-dimensional gradient refractive index (GRIN) micro-optics [29]. Moreover, coupled PSi-DBR systems have been investigated as reliable platforms for chemical and biological sensing [30,31,32,33,34,35], due to the highly sensitive optical response to micro-pores infiltration. In this framework, PSi offers several advantages, especially due to low cost massive production and its tunable physico-chemical properties. Indeed, PSi can be made by an easy and cost effective process based on a computer controlled electrochemical etch of Si wafers in HF/EtOH electrolyte [36]. 

In recent years hybrid plasmonic-photonic systems have been demonstrated as an effective means to obtain impressive field enhancement at subwavelength scales [37,38,39,40,41,42,43]. The basic idea is to bridge the plasmonic and photonic world in order to combine the low mode volume of plasmonics with high Q factor of photonics to produce high local field enhancement compared to the split optical structures. This kind of systems is a promising solution for tailoring the light-matter coupling [44,45,46], for enhancing the magneto-optic activity [47], for modifying the spontaneous emission [48,49,50] and for sensing application [51,52,53]. 

In this line, Badugu et al. [48] proposed to engineer the radiative decay of a fluorophore through a hybrid plasmonic–photonic structure constituted by a metal film layer grown on the top of a DBR. Moreover, they suggested to create holes in the metal film in order to enhance the field coupling between the structure and the fluorophore. In this work, we exploit previous know-how to investigate the properties of a new type of hybrid system constituted by a plasmonic nanograting grown onto a PSi-DBR. It is worthwhile noticing that both individual structures are fabricated through low-cost processes, easily scalable to mass production, an important requirement when application-oriented devices are considered. In particular, we have fabricated and characterized in terms of surface morphology and optical response the individual structures (DBR and plasmonic nanograting or plasmonic cavity crystal, PLC), as well as the hybrid system.

## 2. Materials and Methods 

### 2.1. Preparation of Porous Silicon Photonic Crystals

Porous silicon was produced by electrochemical etching of p-type <100> monocrystalline silicon wafers (resistivity 0.05–0.1 Ωcm) in an electrolyte made of hydrofluoric acid (48 wt%) and absolute ethanol in a 1:1 *v*/*v* ratio, as described in previous works [36]. The process was computer-controlled in order to precisely adjust the anodization current and etching time to obtain a PSi layer of specified porosity. To obtain DBRs, alternate layers of low and high porosity (and, consequently, high and low refractive index, respectively) were prepared by switching between low and high values of current density (within the 10 to 150 mA/cm^2^ range), respectively. To further structure the surfaces, colloidal lithography and plasma deposition were used, as previously done for the fabrication of 2D-arranged PSi wells [54]. 

### 2.2. Preparation of Plasmonic Nanostructured Surfaces

The development of the PLCs was investigated according to previously established fabrication technology [18]. Basically, the PLC consists of a 2D plasmonic cavity crystal (PLC), obtained by a process based on colloidal lithography and plasma techniques. The result is a giga-array of gold/dielectric nanocavities, acting as plasmonic nanoantennas. Either a silicon wafer or glass (D263™T eco, Schott AG, thickness 0.55 mm) were used as substrates to calibrate the etching process and to evaluate the optical response of the obtained surface. A colloidal mask with a pitch of 500 nm was used and the samples were etched under the same O_2_ plasma conditions. Samples with different thicknesses of the polymeric layer (50, 100 and 150 nm) were fabricated. The functional layer of plasma polymerized polyacrylic acid (ppAA) was deposited on bare Si wafer or on glass by plasma enhanced chemical vapour deposition (PE-CVD) using acrylic acid (Sigma-Aldrich, Saint-Quentin-Fallavier, France) as the monomer precursor. The PE-CVD system was based on a capacitively coupled parallel-plate configuration, equipped with a RF power supply at 13.56 MHz. It was operated in bias-controlled mode, with a typical bias of 400 V and a pressure of 20 mTorr. Different ppAA thicknesses were obtained by setting different deposition times (approx. deposition rate: 25 nm/min). In the following, we will call Sample 1, Sample 2 and Sample 3 the structures grown with a polymeric layer of thickness 50, 100 and 150 nm, respectively. 

### 2.3. Preparation of Hybrid Plasmonic–Photonic Structures

**Figure 1:**
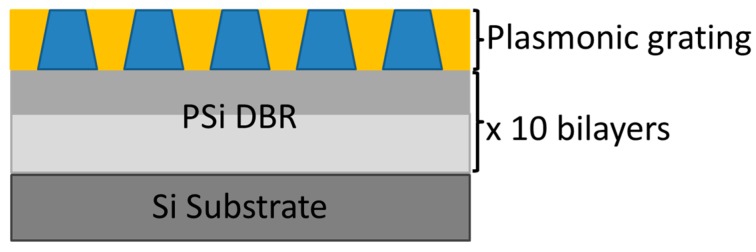
Scheme of the hybrid plasmonic–photonic sample. In the plasmonic grating sketch, the polymeric pillars are colored in blue while the yellow part is gold.

The hybrid plasmonic–photonic systems have been implemented by growing the PLC on top of the PSi multilayer stacks or DBR. Also in this case, structures with three different thicknesses of the polymeric layer (50, 100 and 150 nm) were produced. A scheme of the final sample is given in Figure 1.

### 2.4. Characterization of the Structures

Spectroscopic ellipsometry measurements were performed at three different incidence angles (55, 65 and 75 degrees) in the range 350–1000 nm with a SOPRA ES4G instrument (SOPRA, Bois-Colombes, France) to infer thickness and optical constants of PSi and polyectrolytes (see Section 2.5). 

Scanning electron microscopy (SEM) top view and tilted images of single and hybrid components were acquired by means of a XL30 field emission system (S-FEG, FEI/Philips, Hillsboro, OR, USA). 

Reflectance measurements were carried out in the range of 400–2500 nm with transverse magnetic (TM) and transverse electric (TE) polarized light using a commercial Fourier transform spectrometer (FT-66, Bruker, Billerica, MA, USA) equipped with a home-made variable angle micro-reflectometer. The light source was a halogen lamp while the detection was performed through silicon or InSb photodetectors, depending on the wavelength range of interest. 

Simulations of the DBR were performed with WVASE Software (J.A. Woollam Co., Lincoln, NE, USA) while the simulation of the plasmonic and hybrid structures were performed through a Finite-Difference Time-Domain (FDTD) method with the Lumerical Software (Lumerical solution, Vancouver, Canada). Details on the two simulation methods are reported in Appendix A and Appendix B, respectively.

### 2.5. Polyelectrolyte Probing

Polyelectrolyte solutions of poly(diallyldimethylammonium chloride) (PDDA, Sigma-Aldrich, Saint-Quentin-Fallavier, France) and of poly(sodium 4-styrenesulfonate) (PSS, Sigma-Aldrich, Saint-Quentin-Fallavier, France) diluted at 2% in ultrapure water were used to evaluate the response to the adhesion of nanometric monolayers, of both reference substrates and coupled systems. Thickness and optical constants of the polyelectrolyte layers deposited on plasma polymer or gold reference were inferred by spectroscopic ellipsometry, while the optical behavior due to progressive monolayer adhesion on the PLC and hybrid systems was recorded by means of reflectance measurements.

## 3. Results and Discussion

### 3.1. Porous Silicon Photonic Crystals

Firstly, PSi single layers were produced and investigated. Preliminary simulations of the desired optical properties were introduced taking into account data from literature [55]. Then, several samples were produced using different anodization current densities (10, 50 and 150 mA/cm^2^). Indeed, the formation parameters (current density and etching time) strongly affect the optical constants of the PSi and a proper tuning of the thickness and refractive index of each single layer is fundamental to precisely tune the DBR reflectance peak over a wide range of desired wavelengths. Experimental and simulated optical responses were compared and the predicted analytical model of the PSi layer was refined by inferring thickness and optical constants from the fitting of ellipsometric data. 

**Figure 2:**
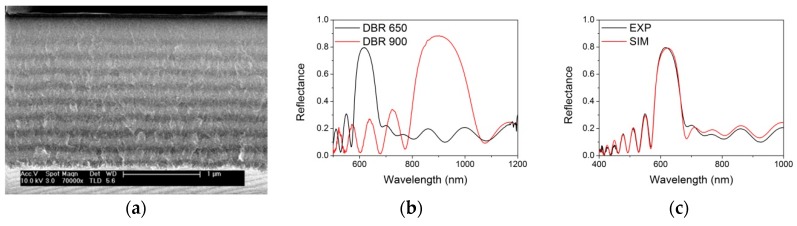
(**a**) Scanning electron microscopy (SEM) image of the cross section of the distributed Bragg reflectors (DBR) centered at 650 nm. (**b**) Reflectance spectrum of the DBR centered at 650 nm (black line) and at 900 nm (red line). (**c**) Comparison between experimental (black line) and calculated (red line) reflectance spectra for the DBR centered at 650 nm.

Distributed Bragg reflectors (DBR) were obtained by alternating the applied current density between two different values, resulting in a multilayer structure. Figure 2a shows a cross section of the DBR.

Different DBRs were specifically designed to show reflectance stop band at different wavelengths in the visible and near-infrared range, as reported in Figure 2b. The DBR centred at about 650 nm consists of 10 bilayers, with alternate layers of high and low refractive index obtained with current densities of 50 and 150 mA/cm^2^, respectively. The total thickness is about 2 µm. The DBR centred at about 900 nm is composed of five bilayers, with alternate layers of high and low refractive index obtained with current densities of 10 and 150 mA/cm^2^, respectively. The total thickness in this case is about 1 µm. Despite the interface in the multilayer structure being not so sharp, the optical response of the DBR is well reproducible and clearly shows an interference pattern (see Figure 2b).

In order to infer the properties of the DBR structure, we have used WVASE software to model the system structure and to calculate the expected optical reflectance. We started from the optical properties and thickness derived from the ellipsometric data of the single PSi layers. The single PSi layers were modelled through an effective medium approximation (EMA) with a certain content of Si, amorphous Si and voids. A fitting procedure of the parameters (EMA contents and thicknesses) allowed for a good reproduction of the optical response of the DBR centred at about 650 nm (see Figure 2c). The details of the layers composition and thickness and the refractive index dispersion are reported in Appendix A.

### 3.2. Porous Silicon Photonic Crystals

**Figure 3:**
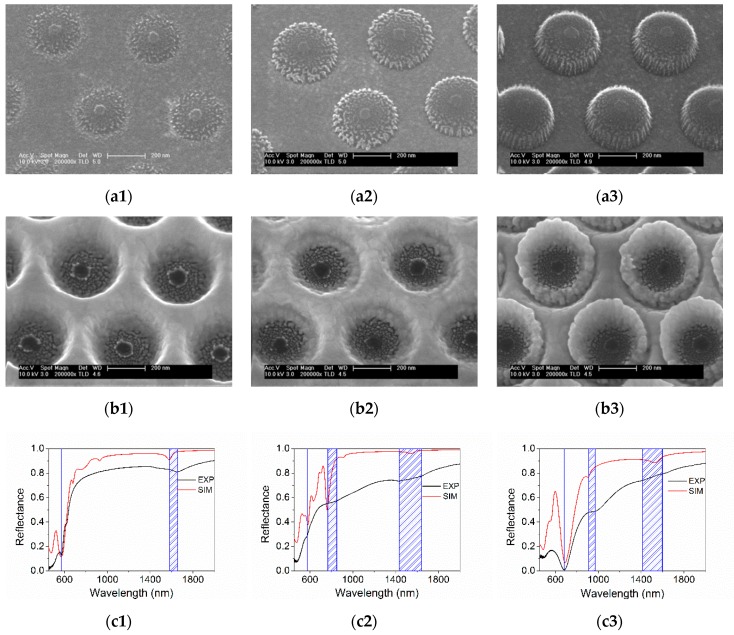
SEM image of the polymeric pillar (**a**) and of the gold nanocavities (**b**) for the Sample 1, 2 and 3, respectively. (**c**) Experimental (black line) and calculated (red line) reflectance spectra for the Sample 1, 2 and 3, respectively. Blue lines and shadowed regions identify the peculiar spectral features.

Figure 3a show SEM images of the polymeric pillars obtained for the different thickness of the PSi-Au interfacing polymeric layer. 

It is evident that a thicker polymeric layer leads to the formation of pillars with a more defined cylindrical shape, while the thinner films lead rather to conical structures. The final structure, obtained after the same nominal deposition of a gold layer (150 nm), are shown in Figure 3b. In the case of smaller polymeric pillars (Sample 1), the dielectric cavities are more pronounced and the pillar top is well immersed in the gold matrix while in the Sample 3 a gold ring is evident on the top of the cavities.

In Figure 3c the black curve shows the near normal reflectance spectra for the three samples. The Sample 1 spectrum shows two dips at about 580 nm and 1600 nm. The spectrum of Sample 2 presents some less defined but evident structures at about 600, 800 and between 1400 and 1600 nm. The reflectance of Sample 3 exhibits a deep minimum around 700 nm and a smaller feature at 900–1000 nm. Small modulations are barely observed around 1400–1600 nm.

The complexity of the structure under study makes the analytical description of the optical response very challenging. Numerical FDTD simulations are helpful to support experimental results and to refine a representative model of the hybrid system, both in a priori and a posteriori approach. We have modelled the three samples assuming the nominal thicknesses for the gold and the polymer layers while the pillar diameter and gold shape have been inferred from the SEM images of Figure 3a. The details of the FDTD model are reported in the Appendix B while the calculated normal incidence reflectance is reported in Figure 3c (red line). As it can be seen from the comparison between experimental and calculated spectra, the simulated spectrum presents more defined and narrow optical structures with respect to the experimental one. On the other hand, for simulation simplicity, regular geometrical shapes with sharp edges have been used in the model whereas in the real samples all the features are quite smooth. Indeed, the real PLC is constituted by a multiplicity of randomly oriented domains which results in a broadening of the experimental spectrum. Moreover, defects and inhomogeneities have to be taken into account, which further contribute to the spectral broadening, preventing a complete agreement with simulations, particularly in terms of absolute intensity. However, the general trend and the relative spectral position of the optical features can be identified in a comparable manner in the experimental- as well as in the simulated-spectra. By analysing the field distribution provided by simulation and on the basis of previous results [18], optical modes can be identified in terms of delocalized and localized plasmonic resonances by analysing the field distribution in the structure at the different resonances. The dip at about 680 nm in Figure 3(c3) can be interpreted as a localized plasmon resonance excited at the top of the pillar. When the pillar dimensions decrease the resonance blue-shifts. The spectral structure at about 1600 nm, which is present in all three samples, may be attributed to the excitation of a delocalized plasmonic resonance at the gold-silicon interface. The other spectral features strongly depend on the shape of the discontinuous gold layer. 

### 3.3. Hybrid Plasmonic–Photonic Structure

**Figure 4:**
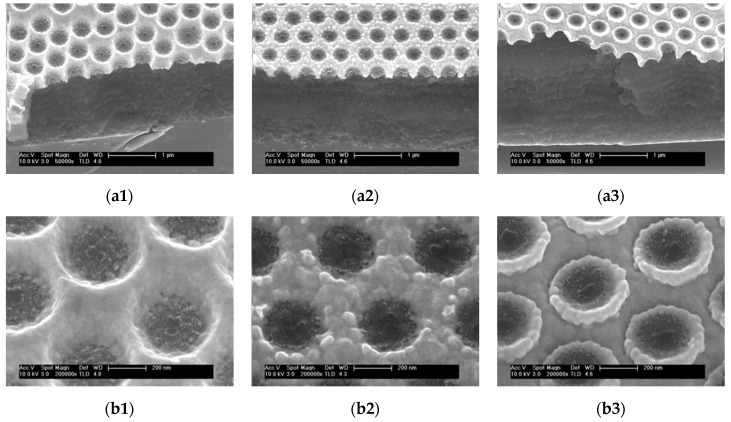

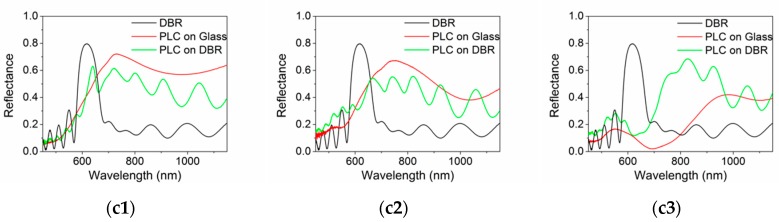
(**a**) SEM images of the hybrid structure for the 1, 2 and 3 polymer layers, respectively. (**b**) Detail of the gold nanocavities for the 1, 2 and 3 polymer layers, respectively. (**c**) Reflectance spectrum of the DBR (black line), of the standard PLC on glass (red line) and of the hybrid structure (green line). As in Figure 3, the numbers 1, 2 and 3 refer to the thickness of the ppAA (50 nm, 100 nm and 150 nm, respectively).

The PLC and DBR systems have been integrated in a hybrid device by growing the plasmonic structure on top of the multilayer stack. In particular, this study refers to PSi-DBRs with a stop band centred around 650 nm. The obtained samples were observed by SEM (see Figure 4a). 

Figure 4b show a detail of the gold nanocavities for the three samples obtained with different polymer thickness. The PLCs grown on the DBR are very similar to those grown on the silicon substrate shown in Figure 3b. 

In order to check the optical coupling in the hybrid structure, we have measured the near normal spectrum for the three samples (see green lines in Figure 4c). For a better interpretation, the reflectance spectra of the hybrid structure, the spectra of the bare DBR (black line) and the ones of the PLC grown on glass (red line) are plotted together. The data of the PLC correspond to samples grown on glass since the average refractive index of the DBR is comparable to that of glass. Apart from a shift in Sample 3 spectrum, the main trend of the spectra of the hybrid samples reproduces those of the PLC grown on glass relatively well, while interference fringes can be observed like in the DBR spectra.

To investigate the interaction effects between the plasmonic and the photonic system we selected Sample 3. In fact, as evident from Figure 4c, this sample presents the best spectral overlap between the plasmonic resonances and the photonic band gap between 600 and 700 nm. 

**Figure 5:**
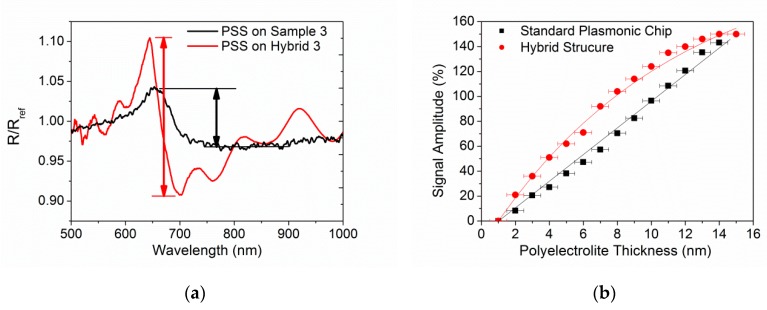
(**a**) Ratio between the reflectance spectra R and R_ref_ acquired for Sample 3 and Hybrid 3 after the deposition of the first PSS and PDDA layer, respectively. The black and red arrow highlight the signal amplitude reported in (**b**) as a function of the polyelectrolyte thickness.

To study the interaction phenomena, we investigated the effect on the reflected light of a change in the refractive index localized at the free surface of the PLC and of the hybrid structure through the progressive adhesion on the chip surface of polyelectrolyte monolayers of known refractive index. Reflectance measurements have been performed with the experimental setup reported in the Materials and Methods Sections. We studied the signal amplitude variation as a function of the polyelectrolyte film thickness in order to evaluate the extension of the plasmonic field above the two surfaces. A drop of polyelectrolyte molecules was deposited on the surface. After five minutes the surface was rinsed with pure water in order to remove residual unbound molecules from the surface and the spectrum was acquired. This procedure was repeated by alternating positively-(PDDA) and negatively-(PSS) charged polyelectrolyte solutions. PDDA and PSS have a nominal refractive index of 1.5 and, due to their charged character, are known to create single monolayers on the surface. The film thickness has been determined by monitoring through spectroscopic ellipsometry the growth of the polyelectrolyte film on gold and ppAA reference surfaces. The film thickness increases by about 1 nm per cycle. Some variance in the thickness increase rate has been registered, particularly for the first polyelectrolyte layer, for which changes in the surface hydrophilicity affect the deposition. Then, we normalized our signal changes with the reflectance after the deposition of the first PDDA layer. Results are shown in Figure 5.

Figure 5a shows the reflectance spectral change related to the deposition of the first PSS layer, either for the standard Sample 3 (polymeric and gold thickness of 150 nm), or for the corresponding hybrid sample. The reflectance ratio is an indicator of sensitivity to refractive index changes and, then, of the local field intensity. One can notice that the hybrid sample exhibits a steeper and narrower spectral shape in the optical signal with respect to the bare PLC. Moreover, the hybrid sample shows an increase in the signal intensity by a factor of about three with respect to the standard sample on glass.

Figure 5b shows signal intensity evolution for successive deposition steps as a function of the polyelectrolyte layer thickness. Whereas for the standard sample the signal increase is almost linear, at least for thicknesses up to 10–15 nm (to be compared with a field extension in the order of 30 nm, as previously measured [19]), for the hybrid sample the signal begins to deviate from a linear behaviour and tends to saturation already for distances lower than 10 nm. However in the explored regime, the signal amplitude registered with the hybrid sample is sistematically higher than that observed with the bare PLC. This result can be interpreted in terms of a redistribution of the electric field. In the hybrid sample, the field is more intense in the proximity of the surface but decays at a smaller distance from it while in the bare PLC the field extends also to relatively larger distances and the system sensitivity keeps a linear trend for progressive changes. Therefore, the hybrid structure is suitable for applications which benefit from a high electric field localized in a small distance range, such as biosensing. Indeed, a limit ascribed to surface plasmon resonance (SPR) is usually the poor sensitivity to small molecules, due to the large field extension (in the order of a few hundreds of nm [56]). A more localized and enhanced field within a few nm above the sensor surface, can be interesting for the detection of small proteins or DNA fragments. Then, the study of this type of system could be promising in the development of a SPR platform with enhanced sensitivity to small molecules or to enhance the fluorescence signal of fluorophore deposited on the top of the system.

In order to better characterize our hybrid system we plan to investigate the angular dispersion of the optical response. This type of analysis could provide a deeper comprehension of the physics of the hybrid systems, highlighting the interplay between plasmonic and photonic modes dispersions.

## 4. Conclusions

We have investigated a flexible, efficient and low cost method to incorporate the plasmonic and photonic systems together. The hybrid structure shows a successful integration of PSi DBRS and gold PLCs in terms of production feasibility and coupling of the optical response. The comparison of experimental data and computational methods allows for an easier analytical description of the complex system. These preliminary results encourage a further study of the hybrid systems, in terms of optical properties manipulation and improvement, as potential platforms for optical filters and biosensors.

## Figures and Tables

**Figure A1:**
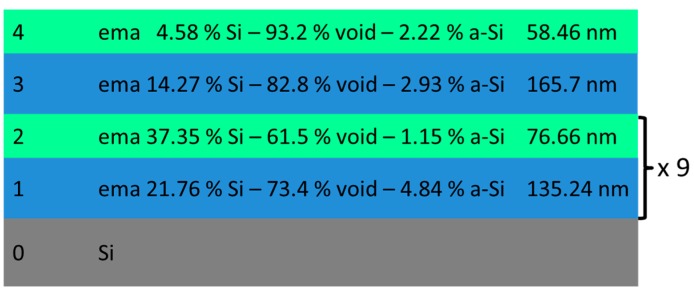
Model of the DBR structure with band gap centered at about 650 nm.

**Figure A2:**
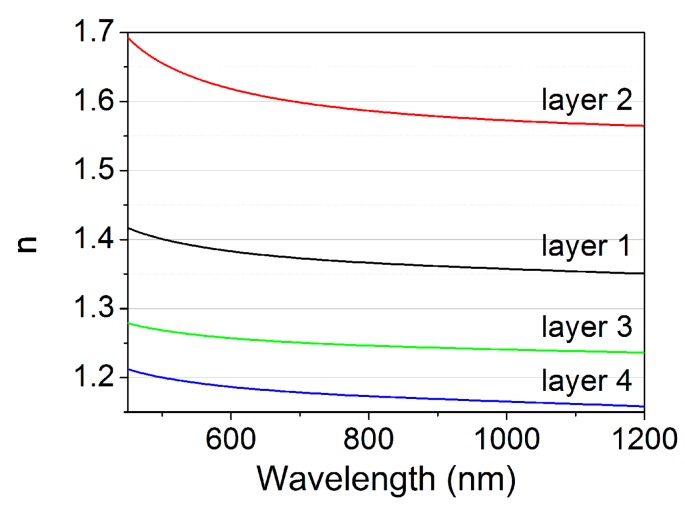
Refractive index n dispersion for the DBR layers reported in Figure A1.

**Figure A3:**
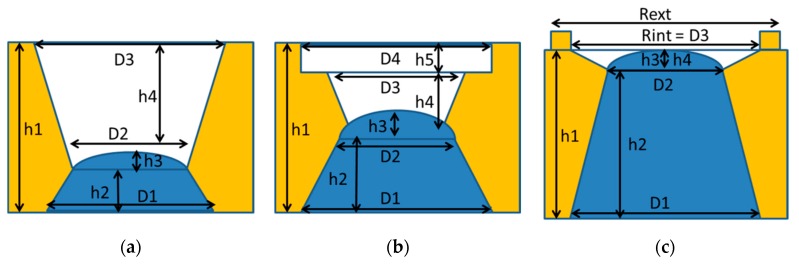
Sketch of (**a**) Sample 1, (**b**) Sample 2 and (**c**) Sample 3 as modelled in the Lumerical FDTD solution software. In each figure the blue shape represents the pillar, the yellow part models the gold while the white region is air. The values of the structures dimensions are reported in Table A1.

## Appendix A

DRB structure has been modelled through the WVASE software (J.A. Woollam Co.). A model of the DBR has been created as the one reported in Figure A1.

The 0 layer is the Silicon substrate (thickness 1 mm). Each layer of the DBR has been modeled through the effective medium approximation (EMA) with a variable content of silicon, void and amorphous silicon (a-Si). A fitting procedure on the reflectance spectrum has been performed in order to optimize the single layer’s composition and thickness. The best configuration is the one reported in Figure A1, with a repetition of nine identical bi-layers and a different structure (composition and thickness) for the two external layers. The optical response is in a very good agreement with the experimental one (see Figure 2c in the main text).

The refractive indexes of the different layers are shown in Figure A2. As evident from the Figure A2, the refractive index of Layer 3 and 4 are very low since the two layer have a very high content of voids. This could be a consequence of the PSi fabrication technique.

## Appendix B

The plasmonic structures were modelled through a Finite-Difference Time-Domain method using Lumerical FDTD Solutions software. All calculations were performed using a unit cell comprising of the elementary cell of the structure and imposing boundary conditions in order to guarantee the periodicity to the system. The mesh size was limited to 5 nm since the nanostructures do not present very sharp structures and there is also some degree of disorder which was taken into account. The plasmonic nanostructures were modeled as a Si substrate overlaid with a 5 nm thick SiO_2_ layer. Each sample was modelled by inferring the pillar and gold shape and dimensions from the SEM images (see Figure 3a). The models for the three samples are reported in Figure A3. In each sketch the pillar is represented by the blue shapes, the gold by the yellow part while the white region is air. Si, SiO_2_ and gold data were taken from Lumerical database.

**Table A1:** Values of the model parameters for the three samples sketched in Figure A3.

Parameter	Sample 1	Sample 2	Sample 3
h1	150	150	150
h2	40	70	110
h3	10	30	40
h4	110	30	40
h5		30	
D1	300	320	340
D2	200	260	260
D3	330	180	340
D4		320	
Rint			340
Rext			420
